# The microbiota-systemic lupus erythematosus axis: mechanisms, diagnostics, and therapeutic frontiers

**DOI:** 10.3389/fimmu.2026.1782828

**Published:** 2026-04-24

**Authors:** Miaomiao Hua, Jun Luo, Pin Li, Yuanyuan Zhang, Xiaqing Zhang, Yu Wu, Hairong Dong

**Affiliations:** 1 Department of Laboratory Medicine, The Fuyang Affiliated Hospital of Anhui Medical University, Fuyang, Anhui, China; 2 Department of Laboratory Medicine, Hohhot First Hospital, Inner Mongolia Autonomous Region, Hohhot, China; 3 Blood Transfusion Department, Ordos Central Hospital, Inner Mongolia Autonomous Region, Ordos, China

**Keywords:** gut microbiota, metagenomics, pathogenesis, precision medicine, systemic lupus erythematosus

## Abstract

Systemic lupus erythematosus (SLE) is a prototypical autoimmune disease in which host-microbiota crosstalk plays a pivotal role in immune dysregulation. Recent metagenomic studies have revealed that disease-specific dysbiosis——characterized by the expansion of pathobionts and depletion of immunoregulatory commensals——occurs across the gut, oral cavity, skin, and genital tract. Integrative multi-omics analyses have identified three mechanistic pathways linking microbial imbalance to autoimmunity: (1) microbial peptides trigger molecular mimicry and epitope spreading, activating autoreactive lymphocytes: (2) microbial metabolites disrupt redox homeostasis, impair epithelial barriers, and skew the AhR-mediated Th17/Treg balance; and (3) dysbiosis alters epigenetic regulation by inhibiting DNA methyltransferases, leading to hypomethylation of SLE-risk genes. Translational studies have shown that microbiome-targeted interventions, including probiotics, prebiotics, fecal microbiota transplantation, and even B cell-depleting chimeric antigen receptor T-cell (CAR-T) therapy, can restore microbial balance, reduce autoantibody levels, and modulate the gut-immune axis. Furthermore, microbial signatures are emerging as potential biomarkers for disease activity and treatment response. Despite this promise, challenges remain, such as the impact of immunosuppressants on the microbiota, spatial heterogeneity in host-microbe interactions, and limitations in causal inference. Looking forward, integrating single-cell metagenomics, microbiota-directed diets, and engineered microbial consortia may pave the way for personalized microbiome-based therapies. Reframing SLE as a “meta-organismal imbalance” positions microbial ecology at the forefront of precision medicine.

## 1 Introduction

### 1.1 Overview of systemic lupus erythematosus

**Figure 1:**
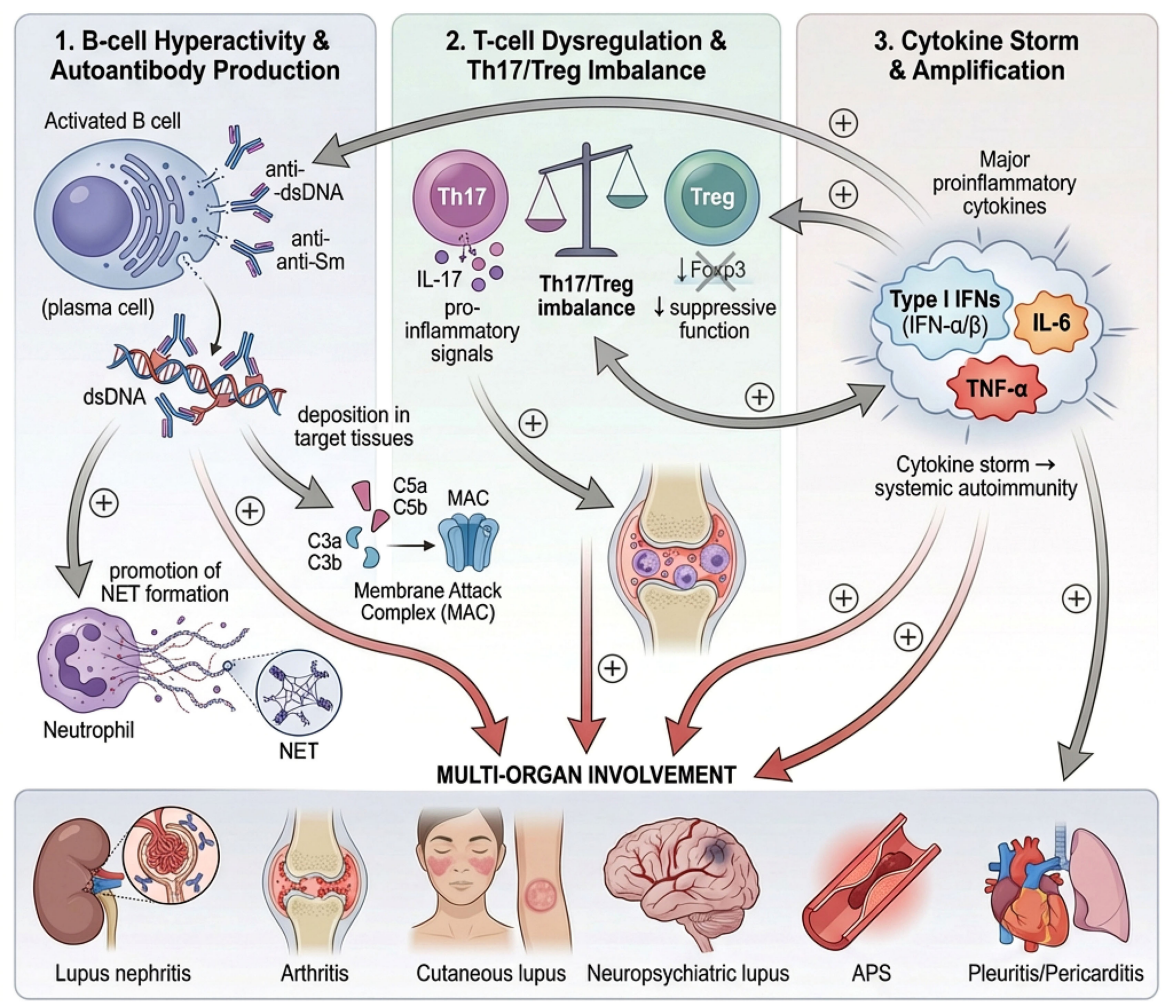
Schematic of the immune tri-axis model in SLE pathogenesis.

Systemic lupus erythematosus (SLE) is a prototypical autoimmune disease marked by widespread immunological dysfunction and multiorgan involvement, particularly affecting the renal glomeruli, synovial membranes, and neurovascular systems (1). At the center of this multisystemic onslaught lies a triad of immune abnormalities. First, B-cell hyperactivity drives the production of pathogenic autoantibodies, such as anti-dsDNA and anti-Sm, which form immune complexes that deposit in target tissues and trigger inflammation—functioning like molecular shrapnel. These immune complexes activate the complement cascade, and promote neutrophil extracellular trap (NET) formation, amplifying local tissue damage (2). Second, T-cell dysregulation further dismantles immune tolerance: Th17 cells promote interleukin-17 (IL-17)-mediated inflammation, while regulatory T cells (Tregs) lose their suppressive function (3). This imbalance ultimately leads to a cytokine storm dominated by type I interferons (IFN-α/β), interleukin-6 (IL-6), and tumor necrosis factor-alpha (TNF-α), converting local inflammation into systemic autoimmunity (**Figure 1**).

The immunological complexity of SLE contributes to its clinical heterogeneity and resistance to conventional therapies. The disease’s persistence is driven by its ability to dynamically respond to environmental cues and epigenetic modifications, rendering it a highly adaptive and resilient disorder (4). For decades, glucocorticoids and broad-spectrum immunosuppressants (e.g., cyclophosphamide, mycophenolate mofetil) have formed the backbone of SLE management, providing a transient reprieve by dampening hyperactive immune responses (5). Nevertheless, these treatments represent a double-edged sword: while effective in controlling acute flares, they fail to eradicate long-lived plasma cells, which reside in survival niches such as the bone marrow and continuously secrete autoantibodies (6). Moreover, chronic corticosteroids use is associated with significant deleterious effects, including osteoporosis, metabolic syndrome, and increased susceptibility to infections (7).

Although targeted biologics—such as those inhibiting B-cell activating factor (BAFF) or type I interferon pathways—have offered improved outcomes for some patients, achieving durable remission remains elusive (8). This therapeutic ceiling underscores the need for a paradigm shift in SLE treatment: one that moves beyond global immunosuppression and instead leverages host–microbiome interactions to restore immune balance. Modulating the microbiota offers a promising path toward recalibrating immune responses without compromising systemic immunity (9).

### 1.2 From bystander to key player: the microbiome’s emerging role in SLE

The etiology of SLE has been attributed to the interplay between genetic susceptibility and environmental triggers (10). Advancements in microbiome research now add a third, equally influential dimension: host–microbe interactions. Alterations in gut microbial composition and function have been associated with the occurrence and severity of SLE manifestations, suggesting a pathogenic role for dysbiosis. This notion is supported by experimental mouse models showing that specific microbiome changes affect disease activity, and further elucidated by MWAS and integrative analysis, which revealed SLE-associated shifts in the gut microbiome and its host interactions (11, 12). For example, specific gut *streptococci*, including *Streptococcus anginosus* and *Streptococcus intermedius*, exemplify this axis, linking microbial metabolic signatures to immune activation in SLE (12, 13).

Technological advances have strengthened these insights. Unlike 16S rRNA sequencing, shotgun metagenomics and clinical mNGS enable species- and gene-level resolution, linking microbial composition with functional pathways (14, 15). Integrated metabolomic and metagenomic analyses suggest that microbial alterations linked to oxidative stress are associated with systemic immune dysregulation (16–19). Emerging single-cell metagenomics further integrates host transcriptomic and microbial datasets, revealing functional interactions such as the gut–liver–immune axis (20). These advances have generated clinically relevant readouts, including microbiota-based activity scores and early trials of targeted probiotic intervention (21, 22).

## 2 Microbiome dysbiosis in SLE: a niche-specific landscape

### 2.1 Gut microbiota: imbalance, loss of tolerance, and pathobiont expansion

Meta-analyses have consistently demonstrated that SLE is associated with significant microbiome disruption. A dedicated SLE-focused meta-analysis including 11 case–control studies confirmed reduced gut alpha diversity in SLE, with significantly lower Shannon index (WMD = −0.22, 95% CI: −0.32 to −0.13) and Chao1 richness (SMD = −0.62, 95% CI: −1.04 to −0.21). Taxonomic analysis further showed decreased *Ruminococcaceae* abundance (SMD = −0.49, 95% CI: −0.84 to −0.15), alongside increased *Enterobacteriaceae* (SMD = 0.45, 95% CI: 0.01 to 0.89) and *Enterococcaceae* (SMD = 0.53, 95% CI: 0.05 to 1.01) (23). A large-scale meta-analysis of 92 observational studies (11,998 participants) reported significantly reduced microbial richness in SLE patients, with a large effect size decrease in Chao1 diversity (SMD = −1.60, 95% CI: −2.54 to −0.66) and a moderate reduction in Shannon index (SMD = −0.63, 95% CI: −1.08 to −0.18). The study further identified shared alterations across rheumatic diseases, including depletion of *Faecalibacterium* and enrichment of *Streptococcus (*24). Extending beyond the gut, a recent meta-analysis across multiple body sites reported reduced alpha diversity and distinct beta diversity in SLE, particularly in gut and oral microbiota (25). Together, these quantitative syntheses provide robust and reproducible evidence linking microbiome dysbiosis to SLE across populations and anatomical niches.

**Figure 2:**
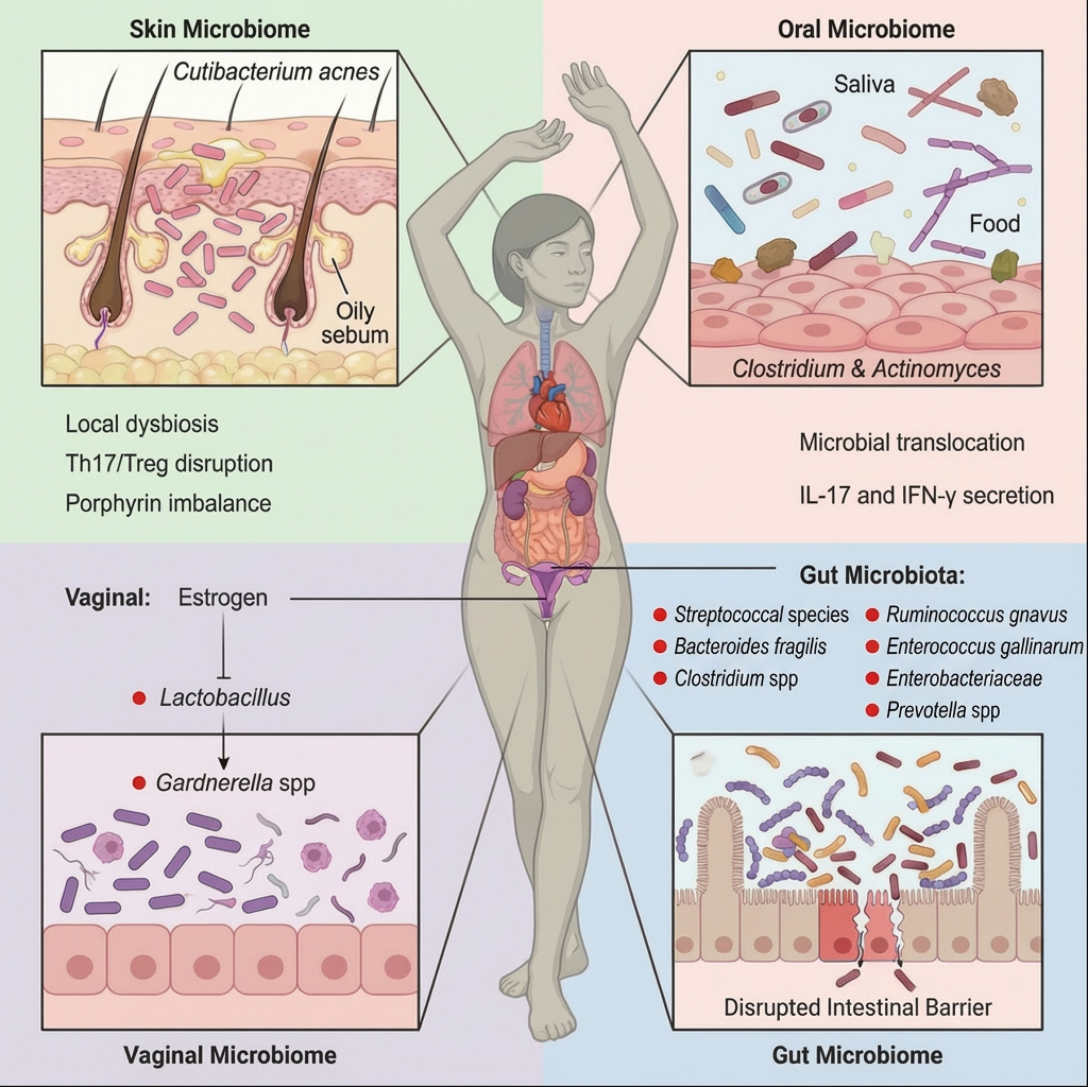
Distribution of multi-site microbiome dysbiosis in SLE.

Metagenome-wide association studies (MWAS) and 16S rRNA sequencing analyses in SLE cohorts consistently demonstrate significant gut microbial dysbiosis at the species level. Increased abundance of *Streptococcus anginosus*, *Streptococcus intermedius*, and related taxa has been reported in patients with active disease, with microbial composition correlating with SLE Disease Activity Index (SLEDAI) scores (12). Broader microbial alterations include reduced levels of the Synergistetes, which is associated with higher anti–dsDNA antibody titers in SLE patients (26). Among the most reproducible findings, *Ruminococcus gnavus* (RG) has emerged as a prominent pathobiont, with marked expansion observed in patients with active lupus nephritis and strong correlations with anti–dsDNA titers and disease activity (27). Serologic profiling further revealed elevated IgG responses against RG cell-wall lipoglycan antigens in SLE patients, linking RG expansion to defined autoantibody subsets (28). Similarly, *Enterococcus gallinarum*, a gut pathobiont, has been identified in the intestinal microbiota of autoimmune patients and shown to translocate to extraintestinal tissues, including the liver, in susceptible individuals. Serologic studies further demonstrated that anti–*E. gallinarum* IgG responses are significantly associated with anti–dsDNA, anti-Sm, and anti–Ribosomal P autoantibodies (29, 30), and IgG3 responses against *E. gallinarum* RNA correlate with disease activity (31). In contrast to these expanded pathobionts, cohort studies consistently report reduced gut microbial diversity in SLE, particularly a decreased relative abundance of *Bifidobacterium*, which negatively correlates with disease activity and inflammatory indices (32). Collectively, these human data indicate that SLE-associated dysbiosis is characterized by expansion of specific immunologically active taxa alongside depletion of potentially protective commensals, reflecting a shift toward a pro-inflammatory microbial ecosystem linked to disease activity and serologic phenotypes. This polymicrobial landscape may foster synergistic pathogenic interactions that amplify autoimmunity (**Figure 2**).

Functionally, gut dysbiosis in SLE involves aberrant metabolic signatures. Sulfur metabolism is disrupted, leading to hydrogen sulfide accumulation, oxidative stress and abnormal autoantigen modification (16, 33). Enrichment of flagellar assembly-related genes suggests enhanced bacterial motility and epithelial penetration. Lipopolysaccharide (LPS) biosynthesis pathways are enriched in SLE-associated microbiomes (see Section 3.2), while branched-chain amino acid (BCAA) synthesis is downregulated (34, 35). Collectively, these functional perturbations underscore the multidimensional role of gut microbial metabolism in SLE pathogenesis.

Recent evidence supports the “oral-gut axis” hypothesis in SLE (36). Strain-level single-nucleotide polymorphism (SNP) analysis revealed genetic identity between *Clostridium* and *Actinomyces* strains in the oral cavity and gut of SLE patients, indicating microbial translocation (35). This may induce systemic autoimmunity via: (1) molecular mimicry between oral bacterial peptides and autoantigens (e.g., Sm); and (2) innate immune activation by ectopic bacteria, which provoke IL-17 and IFN-γ secretion by dendritic cells (37, 38). These findings highlight the gut microbiota as a critical driver of immune activation in lupus (**Figure 2**).

### 2.2 Extraintestinal niches: skin, vaginal, and other microenvironments

Beyond the gut, extraintestinal mucosal and barrier sites also exhibit SLE-associated microbial alterations. The skin microbiome is influenced by UV exposure, which disrupts epidermal immunity and alters protective commensals such as *Cutibacterium acnes* (**Figure 2**) (39–42). These changes are associated with local immune activation and may contribute to cutaneous lupus manifestations. Emerging microbiome-based dermatologic interventions have shown preliminary potential in restoring microbial balance, although their clinical relevance in SLE remains under investigation (43).

The vaginal microbiota also reflects SLE-specific alterations, particularly under estrogen influence, as shown in **Figure 2**. Epidemiological data reveal a striking female predominance in SLE, with women accounting for approximately 90% of cases, particularly during their reproductive years, when disease activity often fluctuates with estrogen levels (e.g., during pregnancy or menstrual cycles) (44). Beyond its direct immunomodulatory effects, including B-cell activation and autoantibody production, estrogen profoundly influences mucosal immunity by altering vaginal microbiota composition. Notably, estrogen reduces *Lactobacillus* dominance and compromises local immune tolerance (45, 46). Emerging evidence suggests that genital tract dysbiosis in SLE patients, characterized by the expansion of opportunistic pathogens such as *Gardnerella* spp., may trigger cross-reactive immune responses through molecular mimicry, thereby exacerbating systemic autoimmunity (46). Furthermore, estrogen metabolites (e.g., 16α-hydroxyestrone) drive LPS production by both the gut and reproductive tract microbiota, activating systemic inflammation via the “gut-genital-immune axis” (47). This mechanistic insight helps explain why exposure to exogenous estrogen (e.g., oral contraceptives) can worsen SLE progression. Conversely, interventions that target genital microbiota restoration (e.g., probiotic supplementation) or aberrant estrogen signaling could offer novel therapeutic strategies for mitigating disease activity in female SLE patients.

### 2.3 Microbial metabolites as immune modulators in SLE

Microbial metabolites such as taurine and short-chain fatty acids (SCFAs) play immunoregulatory roles in SLE. Taurine deficiency is associated with CD4+ T cell hyperactivation via mTORC1 signaling, contributing to immune dysregulation in SLE (48). Taurine supplementation restores immune equilibrium, reduces anti-dsDNA titers, and ameliorates nephritis in lupus-prone mice. Notably, taurine metabolism is tightly regulated by gut microbes, linking microbial ecology to systemic immune tone (49).

Short-chain fatty acid (SCFA)—particularly reduced acetate, propionate, and butyrate—impairs epithelial integrity and enhances microbial translocation (50–52). SCFAs deficits correlate with proinflammatory cytokine profiles (upregulation of IL-6 and TNF-α, downregulation of IL-10) in SLE. Mechanistically, SCFAs modulate immunity via G-protein–coupled receptor activation and histone deacetylase inhibition, modulating adaptive immune polarization (53, 54). SCFA deficiency also depletes the mucosal barrier, facilitating colonization by pathogens such as *Escherichia coli*. Preclinical evidence firmly supports SCFA-based interventions—including probiotic supplementation (e.g., Akkermansia, Lactobacillus) and fiber-rich diets such as resistant starch-that restore gut integrity, reduce renal IgG deposition, suppress the pathobiont *Lactobacillus reuteri* in terms of its abundance and translocation, and ameliorate TLR7-dependent systemic autoimmunity in lupus-prone models via inhibiting plasmacytoid dendritic cell accumulation and type I interferon signaling (55). While these core effects and regulatory mechanisms are well validated by animal experimental data, preliminary human clinical research has provided indirect correlative evidence for the potential value of such interventions in SLE: clinical studies have found that the abundance of Lactobacillus genus is significantly elevated in active SLE patients and correlated with disease severity (56), and microbiota regulation strategies that increase intestinal SCFA-producing bacteria can reduce SLE disease activity scores and pro-inflammatory factor levels. However, direct, large-sample, and long-term follow-up clinical trials specifically targeting SLE to verify the regulatory effects of probiotic supplementation or resistant starch intake on *Lactobacillus reuteri* abundance, renal IgG deposition, and TLR7 signaling pathways remain scarce, and the relevant clinical evidence still needs to be further supplemented and validated.

## 3 Mechanistic insights: how microbiota contribute to lupus pathogenesis

### 3.1 Molecular mimicry and epitope spreading

**Figure 3:**
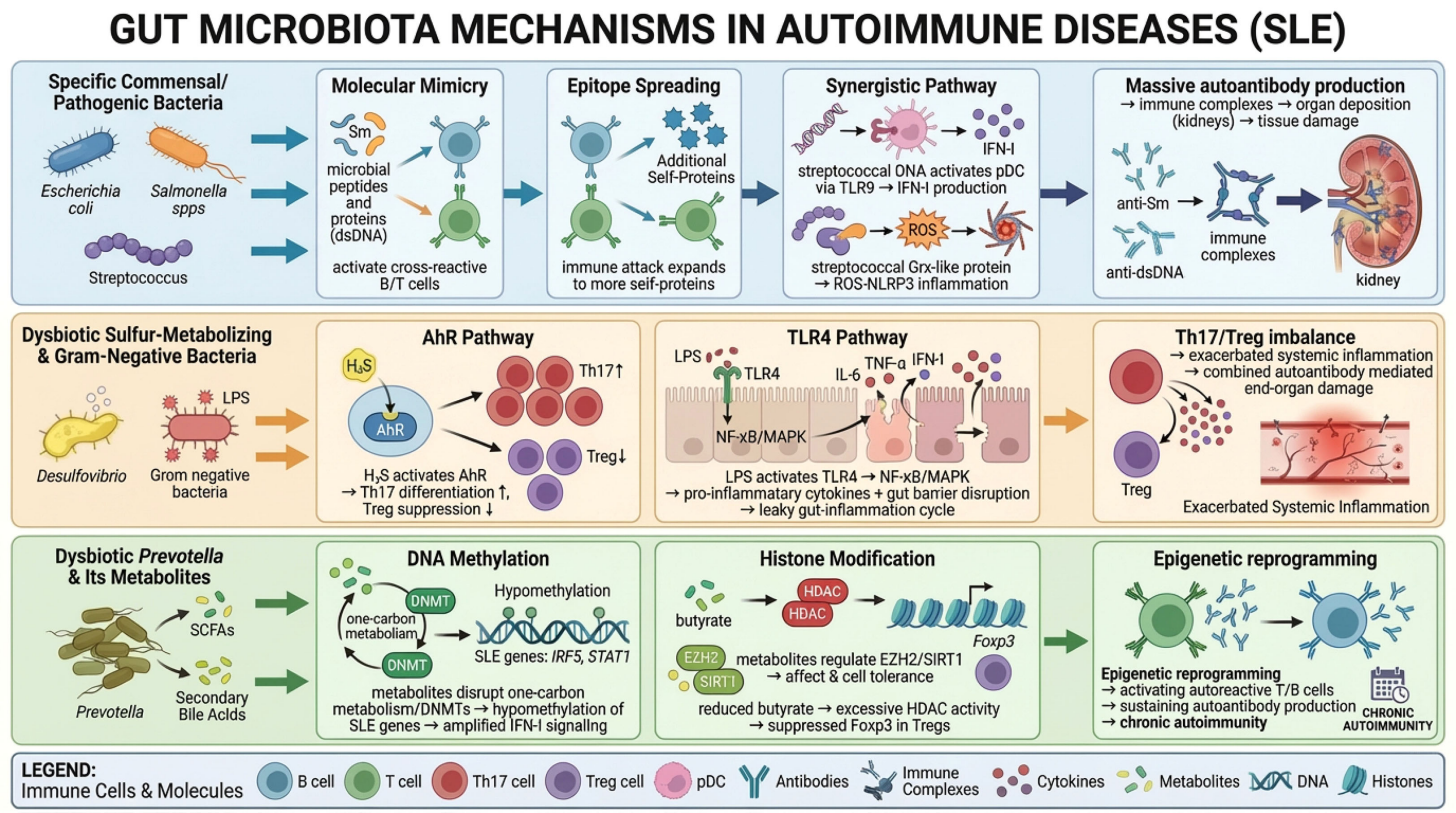
Schematic of the “microbiota-immune-host” tripartite axis model.

As shown in **Figure 3**, the remarkable similarity between gut commensal-derived peptides and SLE-associated autoantigens, as evidenced by molecular mimicry, offers a compelling rationale for the role of microbiota in the pathogenesis of autoimmunity (57). In *Streptococcus pneumoniae*, systematic mapping of bacterial proteins has revealed shared peptide motifs with human proteins, suggesting potential cross-reactive epitopes relevant to autoimmune disorders (58). Similarly, human microbiome–derived peptides structurally analogous to myelin autoantigens have been shown to activate autoreactive CD4^+^ T cells and exacerbate experimental autoimmune encephalomyelitis via MHC II–TCR–dependent molecular mimicry (59). In the context of systemic lupus erythematosus, expansion of *Escherichia*—particularly *E. coli*—has been associated with disease activity, and microbial epitopes derived from these bacteria correlate with autoantibody production and lupus severity (57). Notably, *E. coli* produces curli amyloid fibers that complex with extracellular DNA (curli/eDNA), forming structures that resemble host nucleic acid–containing immune complexes. These complexes cross-react with anti–dsDNA autoantibodies and activate innate immune receptors such as TLR2 and TLR9, thereby enhancing type I interferon signaling and systemic inflammation. Experimental studies further show that exposure to curli-producing *E. coli* or persistent bacteriuria aggravates autoantibody production and lupus-like pathology in murine models (60–62). Together, these findings indicate that *E. coli* may promote lupus pathogenesis through structural cross-reactivity between bacterial DNA-containing complexes and host autoantigens, amplifying autoimmune responses.

Collectively, these findings support a model in which specific bacterial taxa, rather than commensal protective species, may promote autoreactive immune responses through epitope similarity and immune cross-activation. The immunological consequences extend beyond initial cross-reactivity through epitope spreading, in which immune responses originally directed against microbial antigens progressively diversify to target self-proteins, creating an amplifying cascade of autoimmunity (63, 64). Clinical cohort studies have shown elevated serum antibody titers against these bacterial peptides in patients with SLE, with a strong positive correlation to disease activity indices, such as the SLEDAI (57, 60, 65). Importantly, this mimicry mechanism appears to operate synergistically with increased intestinal permeability and impaired antigen presentation by dendritic cells, collectively amplifying the loss of immune tolerance (66).

While these molecular and immunologic pathways provide a conceptual framework, experimental lupus models have further established causal links between dysbiotic microbes and autoimmune amplification. Colonization of lupus-prone mice with *E. gallinarum* resulted in bacterial translocation to the liver and systemic tissues, accompanied by increased autoantibody production and T-cell activation (29). Targeted antibiotic treatment or vaccination against *E. gallinarum* reduced pathogenic immune responses, supporting a causal contribution of this pathobiont to systemic autoimmunity (29). Similarly, gnotobiotic mouse models colonized with lupus-derived RG strains exhibited increased intestinal permeability and augmented anti–dsDNA responses compared with strains derived from healthy donors (27, 28). These findings indicate that strain-specific microbial exposure can amplify lupus-relevant autoantibody production and immune activation in genetically susceptible hosts. Together, these animal studies provide mechanistic validation that dysbiotic microbes are not merely correlated with disease activity, but can actively promote autoimmune responses under permissive host conditions.

### 3.2 Metabolic reprogramming and immune cell dialogue

As shown in **Figure 3**, gut microbiota-derived sulfur metabolites, such as hydrogen sulfide and indoxyl sulfate, play a pivotal role in SLE pathogenesis by mediating metabolic reprogramming and the functional polarization of CD4+ T cells via the aryl hydrocarbon receptor (AhR) pathway (12). Recent findings indicate a notable increase in the abundance of sulfur-metabolizing bacteria, particularly *Desulfovibrio* spp., in SLE patients. These bacteria produce metabolites that activate AhR signaling, promoting the differentiation of proinflammatory Th17 cells while inhibiting the development of Tregs (67, 68).

Mechanistically, AhR activation upregulates the key transcription factor RORγt, which drives excessive production of IL-17 and IL-22, exacerbating systemic inflammation and end-organ damage (69). This pathogenic cascade has been validated in lupus-prone mouse models, where AhR antagonism significantly reduces serum IL-17 levels and ameliorates renal pathology (70). In parallel, microbiota-targeted intervention studies further support a metabolite-driven immune regulatory axis in lupus. Supplementation with Bifidobacterium in lupus-prone mice reduced anti–dsDNA antibody titers, restored Th17/Treg balance, and alleviated renal inflammation. Notably, administration of Bifidobacterium-derived metabolites and SCFAs reproduced these immunomodulatory effects, indicating that microbial metabolic products mediate the impact of Bifidobacterium on adaptive immune polarization (32). Beyond transcriptional regulation, sulfur metabolites may also epigenetically alter immune cell metabolism by modifying DNA methylation, thereby promoting a metabolic shift from fatty acid oxidation to glycolysis. This shift perpetuates the Th17/Treg imbalance (71).

Importantly, the dual nature of AhR signaling contributes to the complexity of this microbial-immune interaction. While most sulfur metabolites exhibit proinflammatory properties, certain ligands such as tryptophan-derived indole-3-carboxaldehyde, have demonstrated anti-inflammatory potential (72). This dichotomy underscores the complexity of microbial metabolic networks and supports the potential of targeted AhR modulation as a precision therapeutic strategy for SLE.

In addition to metabolic reprogramming, pathogen-associated molecular patterns (PAMPs), particularly LPS, play a central role in amplifying inflammatory responses in SLE via activation of the TLR4 signaling pathway (73). In SLE patients, gut dysbiosis—characterized by overgrowth of Gram-negative bacteria such as members of the *Enterobacteriaceae* family—leads to increased LPS production and elevated circulating LPS levels (74). Binding of LPS to TLR4 activates MyD88-dependent NF-κB and MAPK signaling pathways, stimulating monocytes and dendritic cells to produce excessive amounts of proinflammatory cytokines (e.g., IL-6, TNF-α) and type I interferons, thereby initiating a systemic inflammatory cascade (75). Clinical studies have demonstrated significant positive correlations between serum LPS levels and both disease activity (e.g., SLEDAI scores) and anti-dsDNA antibody titers. Furthermore, polymorphisms in the TLR4 gene have been linked to disease susceptibility (76). Mechanistically, LPS contributes to SLE pathogenesis through multiple pathways: (1) it disrupts intestinal barrier integrity, promoting bacterial translocation and perpetuating a “gut leakage-inflammation-autoantigen exposure” cycle (74); and (2) it forms immune complexes with autoantigens such as nucleosomes, which co-activate B cell receptors and TLR4, thereby driving excessive autoantibody production (77).

Notably, animal studies have shown that therapeutic interventions targeting this axis—such as TLR4 inhibitors (e.g., TAK-242) and probiotic administration—can significantly reduce renal IgG deposition and proteinuria in lupus-prone mice (78, 79). These findings provide compelling experimental evidence supporting the LPS-TLR4 axis as a promising therapeutic target in SLE.

### 3.3 Microbiota–epigenome interactions and autoimmune gene derepression

Mounting evidence suggests that the gut microbiota significantly influences SLE progression by modulating host DNA methylation patterns, thereby altering the expression of SLE susceptibility genes (**Figure 3**) (80). Clinical studies have identified a strong association between gut dysbiosis in SLE patients and aberrant hypomethylation of key immune-related genes, including IRF5, STAT1, and TNFAIP3 (80, 81). Overgrowth of *Prevotella* species has been shown to disrupt host one-carbon metabolism via the production of specific metabolites such as short-chain fatty acids and folate (82). This disruption leads to reduced methylation at the IRF5 promoter, enhancing its transcription and intensifying type I interferon signaling pathway (83). This mechanism has been observed in more than 80% of SLE patients and is considered a hallmark feature of the disease (84, 85).

Additionally, microbiota-derived inhibitors of DNA methyltransferases (DNMTs), particularly secondary bile acids, have been shown to directly suppress DNMT activity, contributing to genome-wide hypomethylation (86). Tissue-specific differences along the gut–immune axis have also been observed, suggesting that distinct microbial communities differentially shape methylation profiles in intestinal and peripheral immune compartments (80).

Animal studies provide causal validation of this axis. Fecal microbiota transplantation (FMT) from SLE patients into germ-free mice reproduces key hypomethylation signatures in immune cells, including reduced methylation at IRF5 and STAT1, thereby establishing a direct microbiota-driven epigenetic effect (80). Notably, antibiotic treatment can reverse these epigenetic changes, demonstrating the reversible nature of microbiota-mediated modifications (87).

In addition to DNA methylation, microbial metabolites influence the SLE epigenome by modulating histone modifications (88). Butyrate, a key short-chain fatty acid produced by microbial fermentation of dietary fibers, functions as an epigenetic regulator by inhibiting histone deacetylase (HDAC) activity (51). This inhibition enhances histone acetylation at genomic loci such as the Foxp3 promoter, promoting Tregs differentiation and immunosuppressive function (89).

SLE patients typically exhibit reduced intestinal butyrate levels, leading to aberrant HDAC hyperactivity. This promotes Th17 responses while suppressing Tregs (51). Butyrate also activates GPR109A receptors, inhibits NF-κB-mediated pro-inflammatory cytokine release (e.g., IL-6, TNF-α), and enhances expression of intestinal barrier proteins (e.g., ZO-1, occludin), thereby reducing systemic antigen exposure (90).

Other microbial metabolites, such as propionate and tryptophan derivatives, also modulate B cell tolerance and plasma cell differentiation through epigenetic mechanisms (91). Specifically, these metabolites regulate enzymes including EZH2 and SIRT1 (92). For example, the tryptophan metabolite indole-3-carboxaldehyde suppresses pathogenic B cell activation and autoantibody production via the AhR-SIRT1 axis (93). Preclinical studies highlight the therapeutic potential of microbial metabolite modulation. Supplementation with butyrate-producing bacteria (e.g., *Roseburia* spp.) or direct sodium butyrate administration significantly restores Th17/Treg balance and improves renal pathology in lupus-prone mice (94, 95).

## 4 Translational perspectives: microbiome-based therapeutic strategies

### 4.1 Microbiota-targeted interventions: probiotics, prebiotics, and FMT

**Figure 4:**
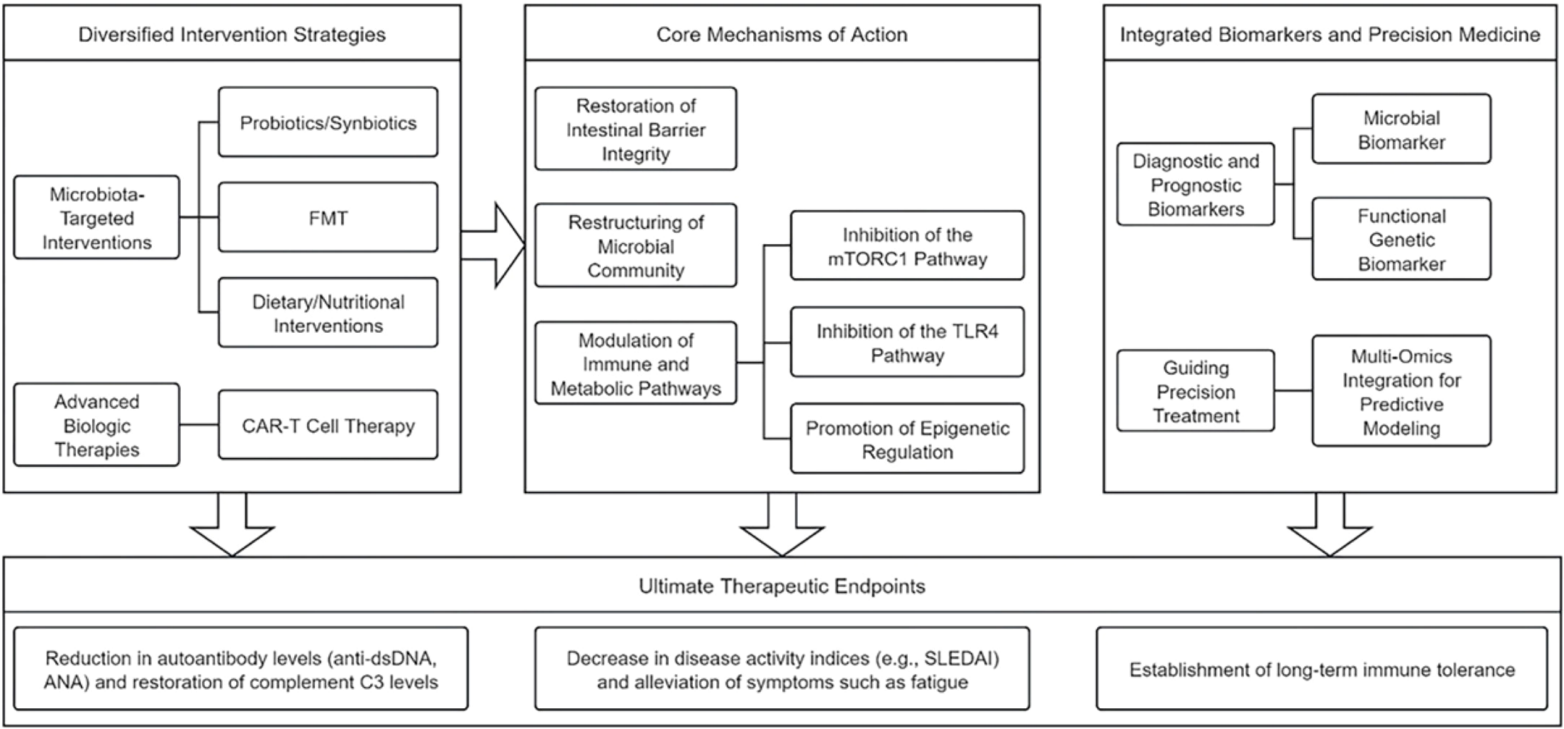
A comprehensive overview of microbiome-targeted therapeutic strategies and integrated biomarkers in SLE.

Combined probiotics-prebiotics therapy reshapes the microbiota and recalibrates immunity in SLE (**Table 1**) (74). Anti-inflammatory commensals such as *Faecalibacterium prausnitzii* restore barrier integrity by producing butyrate and other regulatory metabolites (43). In clinical trials, supplementation with *F. prausnitzii* thickened the colonic mucus layer, lowered serum lipopolysaccharide-binding protein (LBP), and normalized the Th17/Treg ratio (43). Mechanistically, the strain slows lupus-nephritis progression through the activation of the peroxisome proliferator-activated receptor gamma (PPAR-γ) pathway, dampening NF-κB signaling and reducing IL-17 and IFN-α. Multi-strain preparations containing *Lactobacillus* and *Bifidobacterium* species act synergistically: they reinforce epithelial tight junctions and markedly decrease anti-dsDNA antibody titers (**Figure 4**) (26, 96).

**Table 1:** Summary of microbiome-related treatment strategies in systemic lupus erythematosus (SLE).

Intervention methods	Treatment strategies	Mechanism of action
Microbiota-Targeted Interventions	Combined probiotics-prebiotics therapy	a. Anti-inflammatory commensals such as *Faecalibacterium prausnitzii (*43): (1) by producing butyrate and other regulatory metabolites; (2) by thickening the colonic mucus layer, lowered serum lipopolysaccharide-binding protein (LBP), and normalized the Th17/Treg ratio; and (3) through the activation of the peroxisome proliferator-activated receptor gamma (PPAR-γ) pathway, dampening NF-κB signaling and reducing IL-17 and IFN-α. b. Multi-strain preparations containing *Lactobacillus* and *Bifidobacterium* species: reinforcing epithelial tight junctions and markedly decrease anti-dsDNA antibody titers (26, 97).
	prebiotic interventions	selectively promote the growth of *Akkermansia muciniphila*: associating with upregulated expression of tight junction proteins and a decrease in systemic translocation of autoantigens (80).
	dietary interventions	Restriction of aromatic amino acids (e.g., phenylalanine, tyrosine) in diets (99, 101, 102): (1) depriving pathogenic bacteria of essential nitrogen substrates, and (2) attenuating autoreactive B cell hyperactivity through inhibition of the mTORC1 pathway. 94.96.97
	FMT	by increased gut microbiota diversity and decreased abundance of pro-inflammatory taxa such as *Prevotella (*107).
Microbial assisted cell therapy	CAR-T therapy	not only effective depletion of pathogenic B cells but also un unexpected capacity to remodel the gut microbiota-immune axis (110).

Prebiotics offer a complementary route. Preclinical evidence suggests that prebiotic interventions, especially those involving pectin-derived oligosaccharides, selectively promote the growth of *Akkermansia muciniphila* (**Table 1**) (79). This microbial shift has been associated with upregulated expression of tight junction proteins and a decrease in systemic translocation of autoantigens. Collectively, these findings support the potential of targeting the gut-immune axis as a novel therapeutic strategy in the management of SLE.

Beyond direct probiotic supplementation, dietary interventions are emerging as effective strategies for SLE management by modulating microbial metabolic niches (**Figure 4**) (97). Restriction of aromatic amino acids (e.g., phenylalanine, tyrosine) in diets has been shown to inhibit the proliferation of LPS-producing pathobionts (e.g., *Enterobacteriaceae*) while promoting colonization by anti-inflammatory commensals such as *Bifidobacterium* spp. (**Table 1**) (98). Clinical studies have reported that SLE patients adhering to specific dietary regimens exhibit a significant reduction in serum levels of phenylalanine metabolites, such as phenylacetic acid, which correlates with decreased disease activity scores measured by the SLEDAI (99).

Mechanistically, aromatic amino acid restriction exerts dual therapeutic effects: (1) depriving pathogenic bacteria of essential nitrogen substrates, and (2) attenuating autoreactive B cell hyperactivity through inhibition of the mTORC1 pathway (**Figure 4**), resulting in a 32% reduction in autoantibody production compared to controls (100, 101). The Mediterranean diet, rich in dietary fiber and omega-3 polyunsaturated fatty acids, has been shown to provide multiple benefits, including the expansion of SCFA-producing microbiota (102). This intervention attenuates TLR4-mediated inflammatory signaling and has been associated with reduced fatigue severity in longitudinal cohort studies (103). Early-phase clinical trials have indicated that personalized nutritional regimens, particularly those guided by microbiota profiling, offer enhanced precision in symptom management (104). These findings highlight the need to integrate microbial enterotyping with dietary interventions to optimize therapeutic efficacy in SLE.

FMT has emerged as a promising intervention for refractory SLE, yet its long-term safety and efficacy require further validation through large-scale clinical trials (105). Early case reports have demonstrated that SLE patients receiving FMT from healthy donors exhibit notable microbial restructuring (**Figure 4**), characterized by increased gut microbiota diversity and decreased abundance of pro-inflammatory taxa such as *Prevotella* (**Table 1**) (106). These shifts in microbial composition have been linked to improvements in clinical biomarkers, including decreased anti-nuclear antibody titers and increased complement C3 levels (106). Mechanistically, FMT appears to recalibrate host immunity by transferring butyrate-producing consortia (e.g., *Roseburia* spp.) and phage communities, thereby suppressing pathogenic type I interferon production in plasmacytoid dendritic cells (107, 108).

However, therapeutic outcomes show considerable interindividual variability, with some recipients experiencing transient disease flares or gastrointestinal disturbances, emphasizing the urgent need for standardized donor screening protocols and optimized transplantation procedures (80, 106). Emerging preclinical studies are now exploring next-generation strategies aimed at improving treatment precision, including genetically engineered microbial consortia (e.g., IL-10-expressing probiotics) and autologous microbiota reprogramming followed by reinfusion (109). These innovations offer promising avenues to reduce the risk of immune rejection while enhancing therapeutic specificity—key steps toward the realization of personalized microbiome-based therapy in SLE.

### 4.2 Microbial assisted cell therapy

The application of chimeric antigen receptor T-cell (CAR-T) therapy in SLE has demonstrated not only effective depletion of pathogenic B cells but also un unexpected capacity to remodel the gut microbiota-immune axis (**Table 1**) (111). A representative example is the CD19-targeted CAR-T product (TyU19), which has been shown to induce profound depletion of CD19+ B lymphocytes, leading to significant reductions in anti-dsDNA and antinuclear antibody titers while restoring immune tolerance (**Figure 4**) (110).

Clinical investigations have reported that refractory SLE patients treated with TyU19 experience significant changes in gut microbial composition following B cell ablation. These changes include reduced abundance of pro-inflammatory taxa (e.g., *Streptococcus*) and increased prevalence of SCFA-producing genera such as *Roseburia* and *Faecalibacterium (*112). This microbial restructuring may act synergistically with CAR-T-induced immune resetting via two primary mechanisms: reducing bacterial translocation and attenuating LPS-triggered, TLR4-mediated systemic inflammation. Moreover, B cell eradication interrupts the self-perpetuating cycle of immune complex deposition and antigen presentation, indirectly enhancing intestinal barrier integrity (113).

Notably, animal models studies have corroborated these findings, showing that elevated levels of anti-inflammatory microbial metabolites (e.g., butyrate) following CAR-T therapy promote Treg expansion and contribute to the establishment of durable immune tolerance (114, 115). These results underscore the dual immunological and microbial impact of CAR-T therapy and its potential to induce long-term remission in SLE.

### 4.3 Microbial biomarkers for diagnosis, prognosis, and therapeutic monitoring

Mounting evidence identifies *Streptococcus anginosus* as a critical microbial biomarker correlated with SLE disease activity, offering novel opportunities for clinical monitoring (**Figure 4**) (12, 116). Multicenter cohort studies have shown a significant enrichment of *S. anginosus* in both the oral and gut microbiomes of SLE patients, with its abundance positively correlating with SLEDAI scores and anti-dsDNA antibody titers (117). The translational potential of microbiome-derived biomarkers has been significantly enhanced by advances in functional metagenomic analyses, including KEGG pathway annotation, which have enabled the construction of molecular frameworks for predicting therapeutic responses in SLE (118). Of particular interest, genomic signatures of LPS biosynthesis pathways are markedly enriched in glucocorticoid-resistant patients, a pattern that may reflect persistent activation of the LPS-TLR4 axis and consequent evasion of NF-κB signaling (119).

Recent advancements in integrative multi-omics approaches have further transformed the landscape of therapeutic prediction. The construction of microbiota-drug interaction networks, which incorporate microbial genetic signatures with host metabolomic profiles, enables probabilistic forecasting of individual treatment outcomes. This multi-omics integration strategy not only improves prognostic accuracy but also provides a mechanistic blueprint for targeted microbial modulation aimed at enhancing therapeutic sensitivity. These insights forge a critical link between microbial ecology and clinical immunology, laying the foundation for precision medicine in the management of SLE.

## 5 Challenges and opportunities

### 5.1 Methodological and technological limitations

Although metagenomics has significantly advanced our understanding of microbial contributions to SLE, distinguishing true causative relationships from confounding factors remains a persistent challenge. The widespread use of immunomodulatory therapies (e.g., glucocorticoids, hydroxychloroquine) and antibiotics in SLE patients profoundly alters the gut microbiota, making it difficult to discern disease-related microbial shifts from iatrogenic changes (120). For instance, hydroxychloroquine has been shown to attenuate the proinflammatory activity of *Prevotella* via suppression of TLR9 signaling, whereas glucocorticoids have been linked to *Candida* overgrowth—both of which may obscure original microbiota-disease associations (121, 122). Furthermore, dietary factors such as high-salt or low-fiber intake independently influence microbial composition and autoimmune responses, adding further complexity in data interpretation (123).

To address these issues, recent studies have adopted longitudinal cohort designs to track microbiota dynamics before and after interventions, alongside Mendelian randomization analyses to prioritize causative microbial strains. However, the pronounced heterogeneity of SLEs poses significant obstacles to identifying universal microbial signatures. While germ-free mouse models with microbiota transplantation partially validate causal pathways, they often fail to recapitulate the complex immune-microbial interactions observed in humans. This discrepancy highlights the urgent need for advanced statistical frameworks capable of integrating multi-omics datasets with humanized experimental systems to identify context-specific microbial drivers.

Technology advances introduced integrative platforms combining single-cell metagenomics with spatial transcriptomics, offering unprecedented resolution to investigate the spatiotemporal dynamics of microbe-host interactions. Traditional metagenomics treats the microbiota as a collective “black box” and does not resolve the spatial localization of microbial taxa across different niches (e.g., gut, oral mucosa, skin) or their site-specific interactions with immune cells (12, 124). In contrast, single-cell metagenomic profiling enables the identification of *Streptococcus* subspecies colonizing renal or cutaneous lesions in SLE patients, and their co-localization with plasma cell infiltration and autoantigen deposition (125, 126). This is complemented by spatial transcriptomics, which reveals microenvironmental gradients of microbial metabolites such as trimethylamine N-oxide (TMAO) around vascular endothelial cells and maps their role in NLRP3 inflammasome activation and vasculitis (127).

Integration of multi-omics data—combining microbiome, metabolome, and epigenome layers—has enabled the identification of key functional modules, such as the synergy between butyrate-producing microbes and host DNA methylation remodeling, with profound therapeutic implications (128). While challenges remain in scaling these techniques and managing their computational demands, these converging technologies represent a paradigm shift in SLE research, moving beyond correlational studies to establish causal mechanisms and uncover spatially resolved, functionally annotated microbial-host networks for therapeutic targeting.

### 5.2 Bridging research and clinical translation: challenges in application and regulation

The emergence of microbiome signatures has profoundly influenced the therapeutic landscape of SLE, driving a shift toward personalized clinical trial design (106). Recent studies have begun to apply the concept of enterotyping—categorizing individuals based on the dominant gut taxa such as *Bacteroides* or *Prevotella*—to develop targeted therapeutic strategies (107, 129). For example, in optimizing CAR-T therapy, patients with elevated *Akkermansia* levels have demonstrated greater tolerance to higher infusion doses due to better gut barrier integrity. Conversely, patients enriched with *Streptococcus* require dose adjustments to reduce the risk of cytokine release syndrome (CRS) (130, 131).

In addition, microbial functional markers—such as genes involved in butyrate synthesis—have emerged as predictive biomarkers for immunosuppressant responsiveness (132). Elevated butyrate levels are associated with enhanced Th17/Treg rebalancing following belimumab treatment and reduced drug resistance (107). Similar stratification principles apply to probiotic therapies, where multi-strain formulations have shown superior efficacy in patients with “anti-inflammatory microbiota deficiency” enterotypes, achieving higher rates of clinical remission (133, 134). These findings position microbiome-guided precision interventions, including microbiota transplantation, as promising candidates for next-generation SLE therapeutics.

For successful clinical translation, it is essential to establish standardized frameworks for integrating metagenomic, metabolomic, and immunophenotypic data. Pioneering multi-omics platforms now enable refined patient stratification by concurrently analyzing (1) gut microbial signatures (e.g., LPS biosynthesis pathway abundance) (119), (2) systemic metabolite profiles (e.g., serum TMAO) (82), and (3) single-cell immune profiles (135). This tripartite strategy effectively identifies high-risk subgroups characterized by hyperactivation of the TLR4/NF-κB axis, thereby informing the targeted application of TLR4 antagonists in treatment-resistant cases (136).

Nonetheless, data heterogeneity remains a critical translational bottleneck. Challenges such as variable sequencing depth, non-standardized metabolite quantification protocols, and batch effects in immune profiling hinder reproducibility. To mitigate these limitations, international consortia are working to develop unified protocols for biospecimen collection, data generation, and machine learning-based analytical pipelines, thereby facilitating the transition from bench to bedside in microbiome-based SLE management.

## 6 Conclusion and future directions

The field of microbiome research in SLE has evolved from the early stage of cataloging descriptive associations to a more advanced phase of uncovering mechanistic insights, marking a transformative shift in our understanding of disease pathogenesis. The integration of advanced multi-omics techniques—including metagenomics, metabolomics, and epigenomics—has been instrumental in elucidating causal relationships between microbial dysbiosis and the immunopathology of SLE. For instance, certain gut commensals (e.g., *Prevotella copri*) have been shown to directly modulate host immune cells through molecular mimicry of autoantigens such as Sm/RNP. Additionally, microbial metabolites like TMAO have been observed to exacerbate vascular inflammation by disrupting endothelial redox balance. These mechanisms have been validated using animal models and *in vitro* immune cell co-culture systems, demonstrating that FMT from SLE donors induces lupus-like phenotypes in recipient mice via Th17 polarization and interferon pathway activation.

This paradigm shift has fostered the development of specific translational objectives, including the precision editing of microbial genes (e.g., CRISPR-based suppression of LPS biosynthesis) and the therapeutic supplementation of beneficial metabolites (e.g., butyrate-enriched formulations). Several of these strategies are now undergoing clinical evaluation, establishing a direct connection between basic discoveries and their practical applications in the management of SLE.

Looking forward, the integration of microbial, immune, and metabolic networks will define the next frontier in SLE therapeutics. Emerging strategies aim to leverage the “microbiome-immune-metabolism” axis to enable personalized interventions. For example, combining CAR-T cell therapy with microbiota-derived adjuvants (e.g., taurine to promote Treg reconstitution), or implementing precision dietary interventions (e.g., low-aromatic amino acid diets to suppress *Enterobacteriaceae* overgrowth), represents a promising strategy. Preliminary clinical trials have yielded encouraging results: patients stratified by microbial features (e.g., high *Akkermansia* abundance) show improved responses to anti-IFN therapies, while FMT from immunologically “balanced” donors has been associated with reduced glucocorticoid dependence in refractory SLE (**Table 2**).

**Table 2:** Preclinical and clinical studies on microbiome alterations in SLE and their associations with disease characteristics.

Study type	Reference	Core microbiome alterations	Associated disease characteristics
Clinical Study	Tomofuji et al. (12)	Significant enrichment of Streptococcus anginosus and Streptococcus intermedius in the gut; dysregulation of microbial pathways related to sulfur metabolism and redox homeostasis	Significantly positive correlation with SLEDAI scores; associated with activation of IFN-α-mediated inflammatory cascade
Clinical Study	Chen et al. (35)	Increased abundance of Bacteroides fragilis and Clostridium spp. in the gut	Positively correlated with serum anti-dsDNA antibody titers
Clinical Study	Ling et al. (46)	Markedly reduced Lactobacillus abundance and enrichment of opportunistic pathogens (e.g., Gardnerella spp.) in the vaginal tract; concurrent dysbiosis in the gut microbiome	Significantly associated with systemic immune profile abnormalities and disease activity fluctuations in patients
Clinical Study	Huang et al. (106)	Increased gut microbial diversity; decreased abundance of pro-inflammatory Prevotella spp.; restored abundance of short-chain fatty acid (SCFA)-producing bacteria	Decreased serum anti-nuclear antibody (ANA) titers, elevated complement C3 levels, and improved clinical disease activity
Clinical Study	Mirfeizi et al. (21)	Increased abundance of anti-inflammatory commensal Faecalibacterium prausnitzii; improved intestinal barrier function	Significantly reduced SLEDAI scores; restored Th17/Treg balance
Clinical Study	Zhang et al. (80)	Remodeled gut microbial structure after FMT	Reversed hypomethylation of SLE-risk genes (IRF5, STAT1); decreased serum autoantibody levels; improved disease activity
Preclinical Study	Guo et al. (79)	Remodeled gut microbiota structure; restored intestinal barrier integrity	Significantly reduced serum anti-dsDNA antibody levels; decreased renal immune complex deposition; ameliorated lupus nephritis pathology
Preclinical Study	Jin et al. (114)	Reduced abundance of pro-inflammatory Streptococcus spp.; significant enrichment of SCFA-producing bacteria (Roseburia, Faecalibacterium)	Achieved profound depletion of pathogenic B cells; sustained reduction of anti-dsDNA antibody levels; restored immune tolerance

Nevertheless, significant challenges remain, including the need for standardization of multi-omics platforms for real-time patient monitoring and the resolution of ethical issues surrounding global microbiota biobanking. To unlock the full therapeutic potential microbiome-based strategies, future efforts must focus on the development of scalable technologies (e.g., AI-driven microbiome diagnostics) and ensure equitable access to microbial therapies, particularly in resource-limited settings. By decoding the dynamic interplay between microbial ecosystems and host immune networks, we now stand at the threshold of a new clinical paradigm—redefining SLE not as an irreversible autoimmune condition but as a modifiable “meta-organismal imbalance”. Within this framework, individualized microbiome engineering may become the cornerstone of disease remission and long-term management.
